# Minutes of the Ninth Annual Meeting of the Ohio Dental College Association

**Published:** 1861-03

**Authors:** J. Taft


### MINUTES OF THE NINTH ANNUAL MEETING OF THE OHIO DENTAL COLLEGE ASSOCIATION.

The Association met according to adjournment, in the College building, at 10 o'clock, A. M., Tuesday, 19th of February, 1861.

The President, Dr. H. R. Smith, in the chair.

Members present:—Drs. Bonsall, H. R. Smith, Jas. Taylor, C. B. Chapman, T. Wood, Joseph Richardson, Samuel Wardle, J. T. Toland, Geo. Watt, J. Taft, H. A. Smith, and five others by proxy.

The Minutes of the last meeting were read.

The following communication was received and accepted:

CINCINNATI, O., Feb. 18th, 1861.

*To the President of the Ohio College of Dental Surgery;*

DEAR SIR,—We hereby resign our memberships of the Faculty of the Ohio College of Dental Surgery.

J. BYRD SMITH,
GEO. WATT,
C. B. CHAPMAN.

On motion of Dr. H. A. Smith—

*Resolved*, That the thanks of this Association be tendered to the retiring members of the Faculty, for the very able and efficient manner in which they have performed their duties.

Dr. Taylor presented to the Association the following suggestions and considerations, which have been discussed by the Faculty, having in view some changes in the Chairs, viz:

FACULTY MEETING, Feb. 16th, 1861.

On motion of Dr. James Taylor, it was

*Resolved*, That the number of Chairs be reduced to five.

On motion,

*Resolved*, That the Faculty suggest to the Board of Trustees a change in the chair of "Chemistry and Metallurgy" to that of "Chemistry and Physiology," and recommend the election of Dr. C. B. Chapman to the same.

*Resolved*, That the Faculty recommend to the Board of Trustees a change of the chair of "Institutes of Dental Science" to that of "Institutes of Medical and Dental Science."

*Resolved*, That the Faculty suggest to the Board of Trustees a change of the chair of "Anatomy and Physiology" to that of "Anatomy and Histology."

*Resolved*, That the Faculty recommend to the Board of Trustees a change of the chair of "Mechanical Dentistry" to that of "Mechanical Dentistry and Metallurgy."

There was considerable discussion in regard to the affairs of the Institution.

Adjourned to meet to-morrow, at 4 o'clock, P. M.

WEDNESDAY, 20th, 4 O'CLOCK, P. M.

Association met according to adjournment.

Dr. James Taylor presented a report of the names of the present stockholders, with the number of shares of stock held by each one. It is as follows:

| Names. | No. of Shares. | Location. |
|---|---|---|
| James Taylor, - - - - | 10 | Cincinnati, Ohio. |
| Chas. Bonsall, - - - | 1 | " " |
| Thos. Wood, - - - - | 2 | " " |
| Geo. Mendenhall, - - | 1 | " " |
| J. B. Smith, - - - - | 2 | " " |
| J. M. Brown, - - - - | 1 | " " |
| Sam'l Wardle, - - - - | 1 | " " |
| H. R. Smith, - - - - | 1 | " " |
| J. Taft, - - - - - - - | 3 | " " |
| Jos. Richardson, - - - | 1 | " " |
| H. A. Smith, - - - - - | 1 | " " |
| John T. Toland, - - - | 1 | " " |
| C. B. Chapman, - - - | 1 | " " |
| A. Berry, - - - - - - | 1 | Raymond, Miss. |
| G. L. Van Emon, - - - | 2 | ———, Tenn. |
| Edward Taylor, - - - | 1 | Cleveland, Ohio. |
| John Allen, - - - - | 3 | New York, N. Y. |
| W. M. Wright, - - - - | 1 | Pittsburgh, Pa. |
| A. S. Talbert, - - - - | 1 | Lexington, Ky. |
| C. W. Spalding, - - - - | 2 | St. Louis, Mo. |
| H. E. Peebles, - - - - - | 1 | " " |
| H. J. B. M'Kellops, - - | 1 | " " |
| Jas. Knapp, - - - - - - | 1 | New Orleans, La. |
| Chas. E. Keels, - - - - | 1 | " " |
| W. S. Chandler, - - - | 1 | Port Gibson, Miss. |
| D. Dougherty, - - - - | 1 | Danville, Ky. |
| M. N. Manlove, - - - | 1 | Logansport, Ind. |
| J. P. Ulrey, - - - - - | 1 | Rising Sun, " |
| J. W. Baxter, - - - - | 1 | Warsaw, Ky. |
| E. Bray, - - - - - - - | 1 | Evansville, Ind. |
| E. A. Hermon, - - - - - | 1 | Nashville, Tenn. |
| M. DeCamp, - - - - - | 1 | Mansfield, Ohio. |
| Geo. W. Keely, - - - | 1 | Oxford, " |
| Jones, White & M'Curdy, - | 2 | Philadelphia, Pa. |
| J. B. Dunlevy, - - - - | 1 | Pittsburgh, Pa. |
| W. R. Webster, - - - | 1 | Richmond, Ind. |

| Names. | No. of Shares. | Location. |
|---|---|---|
| Geo. Watt, - - - - - - | 1 | Xenia, Ohio. |
| James S. King, - - - - - | 1 | Pittsburgh, Pa. |
| G. J. Fredericks, - - - - | 1 | New Orleans, La. |
| A. J. Reeve, - - - - - | 1 | Mount Vernon, Ohio. |
| Edgar Taylor, - - - - - | 1 | Palmyra, Mo. |
| Samuel Griffith, - - - - | 1 | Louisville, Ky. |
| G. B. Minor, - - - - - | 1 | Milwaukee, Wis. |
| W. W. Allport, - - - - | 1 | Chicago, Ill. |
| D. W. Perkins, - - - - - | 1 | Milwaukee, Wis. |
| I. B. Branch, - - - - - | 1 | Galena, Ill. |
| B. B, Ward, - - - - - - | 1 | ———, Ark. |
| J. M. Lewis, - - - - - | 1 | Marion, Ill. |
| Eli Collins, - - - - - - | 1 | Connersville, Ill. |
| J. B. Martin, - - - - - | 1 | Franklin, Ind. |
| W. H. Goddard, - - - - | 1 | Louisville, Ky. |

The Treasurer would submit the following report:

| | |
|---|---|
| Cash received on rent for College, 1859 and '60, and reported on hand at last meeting.................... | $175.50 |
| Cash, balance of rent for 1859 and 1860.............. | 334.50 |
| Cash, of H. A. Smith, on stock...................... | 50.00 |
| Interest of stock per J. M. Lewis.................... | 6.00 |
| | $566.00 |

He has disbursed the following sums:

| | |
|---|---|
| Paid bill for roofing College.......................... | $162,50 |
| Interest on mortgages......................... | 400.00 |
| Putting in gas fixtures........................ | 43.00 |
| Bill for paving................................. | 18.63 |
| Interest on money borrowed to meet extra outlays, | 22.92 |
| | $647.05 |
| Cash received........................................ | 566.00 |
| | $ 81.05 |
| Floating debt reduced last year to $148.77, added to this.. | 148.77 |
| And interest on sums to this date......................... | 17.85 |
| Amount of floating debt now due.................... | $247.67 |

The account for 1860 and 1861 stands as follows:

| | | | |
|---|---|---|---|
| Cr | Rent for College.............................. | | $510,00 |
| Dr. | Interest on Mortgages.................. | $400.00 | |

Leaving $110 to be applied to liquidating of floating debt, which, when paid in, will leave only $137.67.

February 20, 1861. Jas. Taylor, *Treasurer.*

The Dean presented the following report of the Faculty:

The Faculty respectfully present the following report of the session now about to close. The class has been unusually small this session. The causes for this state of things you can probably understand as well as the Faculty.

There are five candidates for graduation, viz: H. D. Ross, of Montreal, Canada; George S. Allan, of Cleveland, Ohio; William Wasson, of Tennessee; Amos Evans, of Hillsboro', O.; L. M. Griffis, of Hamilton, Ohio.

Dr. M. Wells has filled the place of Demonstrator of Operative and Mechanical Dentistry, and to the satisfaction of all concerned; he will probably soon leave College, and it will be necessary to secure the services of another. This is an important position, and one that in filling the Faculty would be much pleased to have the counsels and assistance of the Association. Some change will be made in the Faculty, but as this has come before you in another way, it is deemed unnecessary to refer to it in detail here. The facilities afforded to the class for practical work in the Infirmary and Laboratory have been much greater than at former times.

The following is a list of the operations performed by the class during the session:

INFIRMARY OPERATIONS.

| | |
|---|---|
| Full set on gold | 1 |
| Partial pieces on gold | 2 |
| Full set continuous gum | 1 |
| Full upper " " | 2 |
| Full set on rubber | 1 |
| Full upper sets on rubber | 20 |
| Partial pieces on rubber | 9 |
| Full upper sets on silver | 4 |
| Under set on " | 1 |
| Partial pieces on " | 10 |
| Under sets cheoplasty | 6 |
| Pieces repaired | 4 |
| Pivot teeth | 4 |
| Fillings of gold | 100 |
| " " other material | 50 |
| Cases of salivary calculus treated | 12 |
| Teeth extracted | 400 |

There was a large amount of work presented, that could not be done for want of time. M. Wells, *Demonstrator.*

J. Taft, *Dean.*

On motion,

*Resolved*, That the suggestions submitted by the Faculty to the Association, in regard to the changes in the arrangement of the chairs, be adopted.

On motion,

*Resolved*, That we now proceed to recommend to the Board of Trustees a suitable person to fill the chair of Anatomy and Histology.

During the discussion of which, the Association adjourned to meet to-morrow, at 12 o'clock.

Thursday, Feb. 21, 1861.

Association met according to adjournment. The President being absent, Dr. Richardson, the 1st Vice-President, called the meeting to order.

The Minutes were read and approved.

On motion,

*Resolved*, That the stockholders relinquish the interest on their stock, for three years from this date, for the liquidation of the debt of the Institution.

The Association proceeded to appoint delegates to the National Dental Association, to meet at Cleveland in July next. The following persons were appointed:

Dr. Geo. W. Keely, of Oxford, O.; Dr. Geo. Watt, Xenia, O.; Dr. M. DeCamp, Mansfield, O.; Drs. Joseph Richardson, John T. Toland, H. A. Smith, and H. R. Smith, Cincinnati, O.; Dr. J. W. Baxter, Warsaw, Ky.; Dr. H. E. Peebles, St. Louis, Mo.; Dr. J. S. King, Pittsburgh, Pa.

Proceeded to the election of officers for the ensuing year. The following persons were elected:

*President*, Dr. C. Bonsall; 1*st Vice-President*, Dr. G. W. Baxter; 2*d Vice-President*, Dr. George Watt; *Secretary*, J. Taft; *Treasurer*, Dr. James Taylor.

The President and Secretary were requested to issue certificates to the delegates to the National Association.

The Association proceeded to nominate a person to fill the chair of Anatomy and Histology, when, upon balloting, Dr. W. H. Atkinson, of Cleveland, Ohio, was found to be unanimously chosen, subject to the Board of Trustees for election.

Association adjourned to meet at 10 o'clock, A. M., Tuesday, Feb. 18, 1862. J. TAFT, *Sec'y.*

